# Improved methods for chronic light-based motor mapping in mice: automated movement tracking with accelerometers, and chronic EEG recording in a bilateral thin-skull preparation

**DOI:** 10.3389/fncir.2013.00123

**Published:** 2013-07-25

**Authors:** Gergely Silasi, Jamie D. Boyd, Jeff LeDue, Timothy H. Murphy

**Affiliations:** ^1^Department of Psychiatry, University of British ColumbiaVancouver, BC, Canada; ^2^Brain Research Centre, University of British ColumbiaVancouver, BC, Canada

**Keywords:** cortex, optogenetics, motor mapping, chronic window, mouse, accelerometer, chronic EEG

## Abstract

Optogenetic stimulation of the mouse cortex can be used to generate motor maps that are similar to maps derived from electrode-based stimulation. Here we present a refined set of procedures for repeated light-based motor mapping in ChR2-expressing mice implanted with a bilateral thinned-skull chronic window and a chronically implanted electroencephalogram (EEG) electrode. Light stimulation is delivered sequentially to over 400 points across the cortex, and evoked movements are quantified on-line with a three-axis accelerometer attached to each forelimb. Bilateral maps of forelimb movement amplitude and movement direction were generated at weekly intervals after recovery from cranial window implantation. We found that light pulses of ~2 mW produced well-defined maps that were centered approximately 0.7 mm anterior and 1.6 mm lateral from bregma. Map borders were defined by sites where light stimulation evoked EEG deflections, but not movements. Motor maps were similar in size and location between mice, and maps were stable over weeks in terms of the number of responsive sites, and the direction of evoked movements. We suggest that our method may be used to chronically assess evoked motor output in mice, and may be combined with other imaging tools to assess cortical reorganization or sensory-motor integration.

## INTRODUCTION

Optogenetic stimulation is a well-suited method for studying the motor system in rodents. The availability of multiple lines of mice expressing either excitatory or inhibitory opsins in selective neuronal subpopulations makes it possible to evaluate the contribution of individual cortical points to motor output (Ting and Feng, 2013). During light-based motor mapping (LBMM), the cortical region representing forelimb muscles can be identified by sequentially stimulating different cortical points and recording the evoked electromyographic (EMG) activity from the contralateral forelimb (Ayling et al., 2009; Hira et al., 2009). This method complements previously available tools, such as intracortical microstimulation (ICMS) where an electrode is lowered into deep cortical layers and limb or body movements are evoked through current injection (Asanuma and sakata, 1967; Li and Waters, 1991; Kleim et al., 1998; Monfils et al., 2005; Tennant et al., 2011; Young et al., 2011; Barbay et al., 2012). LBMM offers two distinct advantages over traditional ICMS: the effect of light-based stimulation is more specific as it is restricted to cells expressing the light-sensitive opsin, and light-based stimulation may be performed repeatedly, without the need for a craniotomy. Despite these advantages, relatively few studies have performed longitudinal motor cortex stimulation (Harrison et al., 2012), and none with bilateral stimulation in combination with electroencephalogram (EEG) recording.

One reason for this paucity of studies may be that the chronic windows made by craniectomy are susceptible to bone regrowth, causing a deterioration of window quality often resulting in the exclusion of animals (Harrison et al., 2012). To improve the consistency of longitudinal motor mapping, we modified the “polished and reinforced thinned-skull” (PoRTS) preparation (Drew et al., 2010) by expanding the cranial window to include both hemispheres and combined it with a chronically implanted EEG electrode and a head-fixing assembly. Thinned-skull preparations provide optical clarity sufficient for optogenetic stimulation, and importantly produce less inflammation compared to craniectomy procedures (Grutzendler and Gan, 2006). Using this protocol, we are able to visualize the majority of the dorsal cortical surface of the mouse brain (~20 mm^2^) and evoke motor maps from both hemispheres in ChR2 expressing animals (Thy-1 ChR2, line 18). Light-induced cortical depolarization can be monitored through the chronic EEG electrode, while evoked forelimb movements are quantified on-line with lightweight accelerometers attached to the forelimbs. Using this preparation, we show that LBMM may be performed in parallel with chronic EEG recordings up to 10 weeks post-surgery, thus allowing for the assessment of topographical features of forelimb movement representations and the quantification of cortical depolarization in the mouse brain.

## METHODS

### SUBJECTS AND EXPERIMENTAL DESIGN

All experimental protocols were approved by the University of British Columbia Animal Care Committee. Mice expressing Channelrhodopsin-2 under the Thy-1 promoter [line 18, stock 007612, strain B6.Cg-Tg(Thy1-COP4/EYFP)18Gfng/J] were obtained from Jackson Labs and a breeding colony was established. Adult male mice, weighing approximately 25 g at the time of surgery were used for all experiments.

### SURGERY FOR THINNED-SKULL PREPARATION

A novel bilateral chronic window preparation was developed based on the reinforced thin-skull preparation (Drew et al., 2010). There are no previous reports of chronic preparations that provide bihemispheric optical access combined with chronic EEG recordings in mouse. Prior to starting the surgery, a No. 1 circular cover-glass (Marienfeld, Lauda-Konigshofen, Germany; Cat #: 0111520) was cut with a diamond pen (ThorLabs, Newton, NJ, USA; Cat#: S90W) to the size of the final cranial window (~9 mm diameter). Mice were anesthetized with isoflurane (4% induction, 1.5% maintenance) and fixed in a stereotaxic apparatus. Body temperature was maintained with an electric heating pad servo regulated by a rectal thermometer. Lidocaine (20 μl) was injected under the scalp to reduce discomfort, and dexamethasone (0.2 mg/kg) was injected intramuscularly to minimize any possible inflammation. A skin flap (approximately 6 mm diameter) extending over both hemispheres was cut and removed, and the underlying bone was cleared of connective tissue. A high-speed dental drill (Dentsply, Woodbridge, ON, Canada; Product: Midwest stylus 540s) was used to thin the bone over an area extending ~4 mm anterior and 5 mm posterior of bregma, respectively, and bordered laterally by the temporal ridge. To avoid overheating, drilling was paused intermittently, and the surgical area was covered with room temperature aCSF. When moistened, a properly thinned-skull becomes transparent, therefore allowing one to gage if the area of interest is sufficiently thinned. The bone was thinned until the spongy middle layer, containing vasculature, was completely removed. The skull is significantly thicker around the coronal and sagittal sutures, requiring more thinning in these regions, however, special care must be taken to avoid damaging the superior sagittal sinus when drilling across the midline. Once the bone was evenly transparent the area was rinsed with aCSF buffer to remove any debris, and allowed to briefly dry. To record EEG, the tip of a 0.013-mm diameter stainless steel acupuncture needle (Wellsprings Products, Ferndale, WA, USA; Cat#: 0102114131) was inserted into the epidural space, just lateral to the thinned area at the level of bregma. The needle was advanced through the skull by manually turning it back and forth while applying downward pressure. The tip of the needle was viewed through a dissecting microscope to ensure it was advanced through the bone, but had not damaged the brain. With the EEG electrode in place, a clear-drying dental adhesive (Parkell, Edgewood, NY, USA; Product: C&B Metabond) was applied generously to the area, including the base of the EEG electrode. The pre-cut cover glass was placed on top of the thinned window and pressed flat into the adhesive. A head-fixing screw was added by pressing a 4/40 stainless steel setscrew into the drying dental adhesive, posterior to the chronic window. This setscrew was used in place of ear-bars to stabilize the head during mapping. If necessary, additional dental adhesive was added, to ensure that all of the exposed bone was covered, and that the incision site was sealed at the borders. Once the adhesive had dried, most of the exposed end of the acupuncture needle was cut away with wire cutters, leaving a 3 mm contact above the dried adhesive. In our experience this thinned-skull preparation provided sufficient clarity and optical access without the need for the tin oxide based polishing step (Drew et al., 2010). In addition, we found that replacing the cyanoacrylate adhesive with the Metabond dental cement eliminated the occurrence of highly scattering imperfections that were often seen with cyanoacrylate preparations.

### CHRONIC MAPPING OF MOTOR FUNCTION AND CORTICAL EXCITABILITY

**FIGURE 1:**
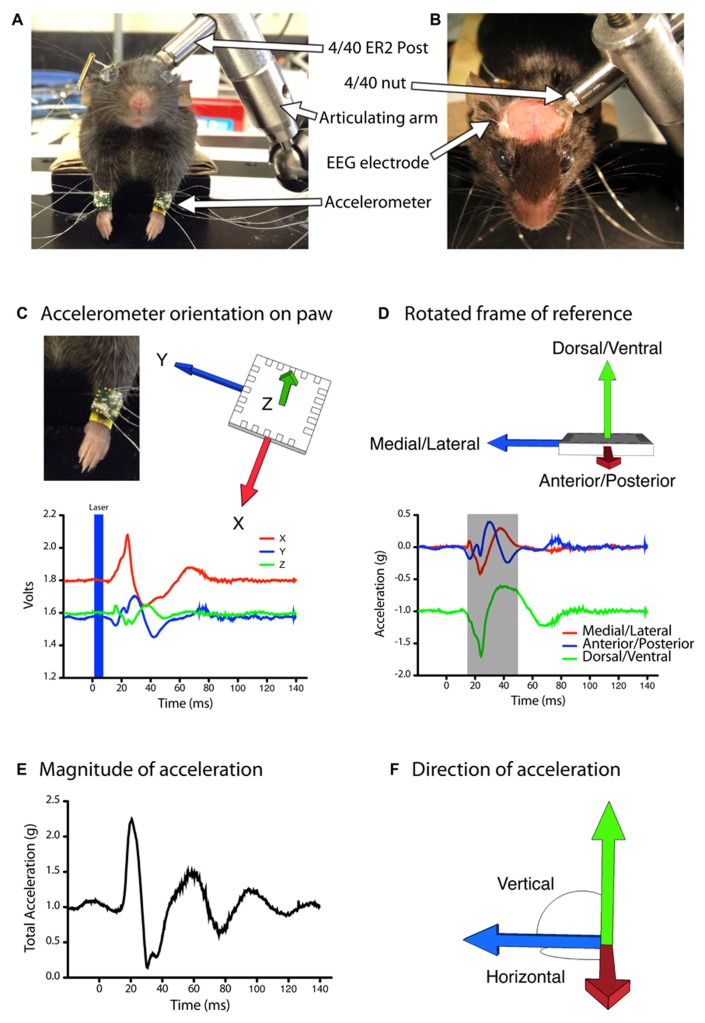
**Automated quantification of evoked movements during light based motor mapping.(A,B)** The head of the anesthetized mouse is stabilized with a head-fixing assembly consisting of a stainless steel (ER2) post, a 4/40 threaded nut and an articulating arm. The mouse is positioned with its limbs freely hanging in a natural posture. **(C,D)** Accelerometers are attached to each forelimb and the analog voltage signals are rotated into a frame of reference that aligns all of the acceleration due to gravity into the vertical (*Z*) axis. **(D)** The magnitude of acceleration averaged over a 30 ms period (gray shaded region) was used to construct motor maps based on peak acceleration **(E)** as well as movement direction in the vertical or horizontal planes **(F)**.

Following chronic window implantation mice were allowed to recover for at least 5 weeks. LBMM was carried out three times for each mouse at weekly intervals, with the exception of two mice, where the last map was generated 1 month after the second map. For each mapping session, mice were anesthetized with an IP injection of ketamine (60 mg/kg) and xylazine 10 mg/kg) and supplemental doses of ketamine alone were delivered as needed to maintain anesthesia. Anesthetic depth was determined by monitoring spontaneous whisking and the toe-pinch withdrawal reflex (Tandon et al., 2007). The mice were placed on a custom made baseplate equipped with an articulating arm (ThorLabs, Newton, NJ, USA; Product: TRB1) for stabilizing the head, and an elevated platform to support the body of the mouse. Body temperature was maintained via a heating pad placed underneath the animal and controlled by a rectal thermometer. The head of the mouse was connected to the articulating arm on the base plate via the setscrew next to the chronic window (**Figures 1A,B**). To achieve greater stability at this connection, a 4/40 nut was first threaded onto the setscrew, followed by a stainless-steel threaded ER2 post (ThorLabs, Newton, NJ, USA; Product: ER2). The nut was then tightened firmly (upward) against the ER2 post (as opposed to tightening downward against the dental cement on the head). This configuration stabilized the head without directly applying torque or pressure.

The body of the mouse was positioned on the elevated platform, with the head raised slightly above the body, and the forelimbs hanging freely over the edge (**Figure 1A**). This position mimics the natural posture of the animal and allows the forelimbs to move freely during evoked movements. A three-axis MEMS accelerometer (ST Microelectronics, Coppell, TX, USA; Cat#: LIS344ALH) was attached to each forelimb with wristbands constructed out of heat-shrink tubing. We selected these accelerometers as the output is an analog voltage signal that can be sampled at high frequency during data acquisition, and the device is highly sensitive to changes in accelerations (0.66–0.22 V/*g*). In addition, the compact size (4 mm × 4 mm × 1.5 mm dimensions) and weight (40 mg) impose negligible constraints on the limb during motor mapping, as mice readily grasp and consume 45 mg food pellets during skilled reaching tasks (Zeiler et al., 2013). Wired connections to the accelerometer were made with 50.8 μm diameter insulated stainless steel wires (A-M Systems, Carlsborg, WA, USA; Cat#: 790500) attached with silver conductive epoxy (MG Chemicals, Cat#: 8331-14G). Wires were cut approximately 5 cm long to allow a full range of motion for the forelimbs. The analog voltage readout from each accelerometer channel was sampled at 5 kHz with custom written Igor Pro software (WaveMetrics) via a NIDAQ 6221 digitizer board. EEG signal was recorded by connecting the implanted EEG electrode and a subcutaneously placed ground electrode in the neck of the animal to a differential amplifier (A-M Systems, Model 1700) set at 1000× gain.

**FIGURE 2:**
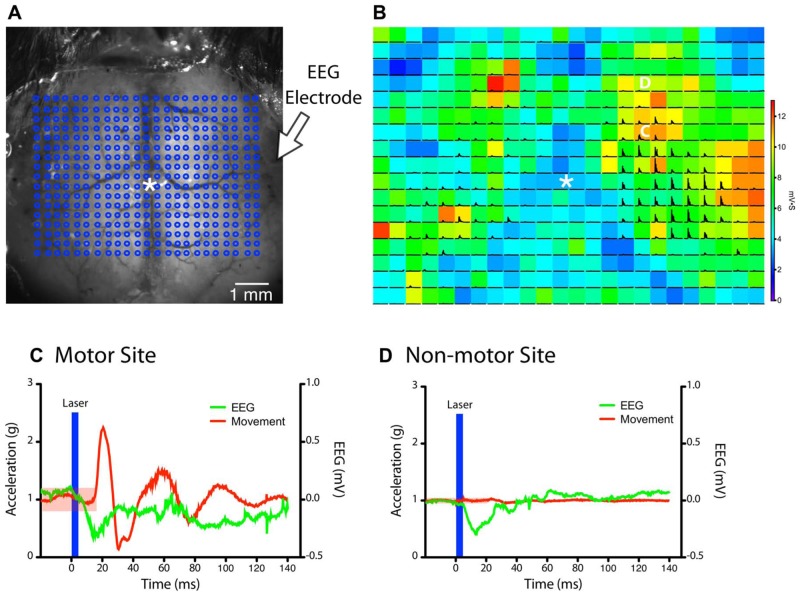
**Simultaneous monitoring of light stimulation evoked forelimb movements by accelerometers and cortical depolarization by EEG.(A)** A grid of stimulation sites (18 × 23 points) within the chronic cranial window is targeted in random order by a collimated laser beam (asterisk “*” indicates bregma). **(B)** The magnitude of the EEG deflection for each pixel is represented as a colored heat-map, while black traces show the magnitude of acceleration recorded from the left forelimb (each pixel = 300 μm). The accelerometer and EEG signals from the points marked by the white C and D are expanded in the panels below. **(C,D)** Example traces of accelerometer (left paw) and EEG signals after stimulation of a site within the motor map **(C)** and outside the motor map **(D)** in the right hemisphere (from **B**). The region shaded in red indicates the threshold for detecting a movement (five times the SD of baseline data). A clearly visible EEG deflection is observed in both examples immediately after the light stimulus (blue bar). The movement response from the motor site **(C)** was delayed by ~18 ms after stimulus onset.

Light stimulation was provided by a 473-nm diode pumped solid-state laser (CNI, Optoelectronics, Changchun, China) targeted to the brain through a custom made macroscope (Ayling et al., 2009; Harrison et al., 2009). To achieve square light pulses, the laser was operated in continuous wave and a Pockels cell controlled by Igor Pro software modulated the power and duration of light pulses. A grid of approximately 18 × 23 stimulation points spaced 300 μm apart was superimposed on an image of the cortex and a 5-ms light pulse was delivered to each point in a random order by moving the mouse underneath the beam with an *x*–*y* stage controlled by Igor Pro software. Initial maps were generated at multiple laser powers delivered in an interleaved fashion (**Figure 2**), and all subsequent maps were derived from a single 2 mW laser stimulus delivered to each site. Laser power was measured at the plane of the brain surface with a powermeter (ThorLabs, Newton, NJ, USA; Product: PM100D).

### MOTOR AND CORTICAL EXCITABILITY MAP ANALYSIS

Maps were generated based on the peak acceleration as well as direction of the evoked movements. At each stimulation site, baseline data was recorded for 0.5 s prior to the light pulse, followed by 1 s of post-stimulus recording. The raw voltage collected from each channel was multiplied by the calibration value provided by the manufacturer (0.22 V/*g*) to convert the voltage into acceleration units (*g*). For each stimulation point, the peak magnitude of the evoked acceleration was determined by summing the values from the *X*, *Y*, and *Z* axes of the accelerometer according to the formula

$$\left|A\right|=\sqrt{A_{x}^{2}+A_{y}^{2}+A_{z}^{2}}$$

where ∣A∣ is the magnitude of the acceleration vector and *A*_x_, *A*_y_, *A*_z_ are the components of the acceleration vector. In order for a signal to be considered an evoked movement, it had to exceed five times the standard deviation (SD) of the baseline data for that stimulation site. This threshold was sufficient to eliminate any small limb movements caused by respiration. Average motor output was calculated for each map by calculating the average acceleration evoked by all active pixels in each forelimb map. The center of gravity was defined as the *X*, *Y* coordinate where the weighted relative amplitude of the total evoked acceleration was equal for all four quadrants of the map.

To calculate a vector for the evoked movements, we integrated the initial 30 ms portion of the acceleration signal from the *X*, *Y*, and *Z* channels (**Figures 1D–F**). Given that there were slight differences in the exact placement of the accelerometer on the forelimb, as well as differences in the resting position of the forelimb before the evoked movements, comparing this raw vector between trials or between animals would be inaccurate. To correct this, we applied a rotation matrix to our data to express limb acceleration vectors relative to the fixed vector of gravity (**Figures 1C,D**). Based on the assumption that prior to light stimulation (with the limb at rest) all of the acceleration signal is due to gravity, we created a rotation matrix to rotate the raw baseline acceleration vector such that the signal due to gravity is aligned with the negative vertical (*Z*) axis. This is also the optical axis of the macroscope. We scaled the magnitude of the baseline acceleration vector to ensure the acceleration due to gravity was -1 *g*. In this orientation accelerations in the *X*-axis indicate movements in the anterior/posterior direction, *Y*-axis accelerations indicate medial/lateral movements, and *Z*-axis accelerations indicate dorsal/ventral movements. To represent forelimb movement direction topographically across the brain we generated color-coded maps based on the angle of limb acceleration within the horizontal plane (M–L/A–P) and out of the horizontal plane, i.e., elevation (D–V) according to the following formulas (**Figure 1F**):

$$\begin{array}{c} \tan(horizontal\,\;plane)=A_{y}^{\prime }/A_{x}^{\prime }\\ \tan(elevation)=A_{z}^{\prime }/\sqrt{\left(A_{x}^{\prime 2}+A_{y}^{\prime 2}\right)} \end{array}$$

where $A_{x}^{\prime },A_{y}^{\prime },and\,\,\,A_{z}^{\prime }\;$ are the components of the acceleration vector after rotation.

Electroencephalogram maps were generated by integrating the EEG signal for 70 ms after the light pulse at each stimulation site. To account for spontaneous EEG fluctuations, spatial averaging was performed across the nine pixels surrounding the stimulation site. As a consequence, it is possible that some of the signal in an active pixel of the EEG map receives partial contribution from activity in a neighboring pixel. An alternative to spatial averaging is to stimulate each site multiple times to generate more stable baseline EEG data, however, we chose to do spatial averaging to avoid any possible potentiating effects of repeated stimulation. For quantification of EEG maps the integrated value for each point was divided by the integration period.

## RESULTS

### STABILITY OF CHRONIC WINDOW PREPARATION

We found that the mice tolerated the bilateral cranial windows without noticeable discomfort or health complications, similar to the original PoRTS preparation (Drew et al., 2010). Post-surgical mice can be group housed following full recovery from anesthesia. All five mice that were mapped in this study maintained viable chronic windows, with surface blood vessels clearly visible for at least 3 months post-implantation. No animals were excluded due to inadvertent window detachments or other technical failures.

### QUANTIFYING EVOKED MOVEMENTS WITH ACCELEROMETERS

We first determined whether forelimb movements could be reliably quantified with accelerometers during LBMM (**Figure 2A**). Converting the voltage readings from all three axes to acceleration (in *g*) showed that evoked movements typically started 18 ms after the onset of the laser stimulus (**Figure 2C**), and ranged in amplitude from ~0.5 to 5 *g*, depending on the site of stimulation and the level of anesthesia. EMG responses also occur at a similar latency after light stimulation (Ayling et al., 2009). Simultaneous EEG recording allowed us to differentiate between points with both light-evoked movement and EEG deflection versus points that produced an EEG deflection but no movement (**Figures 2B–D**). Although surface blood vessels do not appear to influence the magnitude of cortical depolarization, stimulating directly over the superior sagittal sinus often did not produce an EEG response due to significant light absorption and minimal penetration of light to neighboring photoexcitable cortical tissue.

**FIGURE 3:**
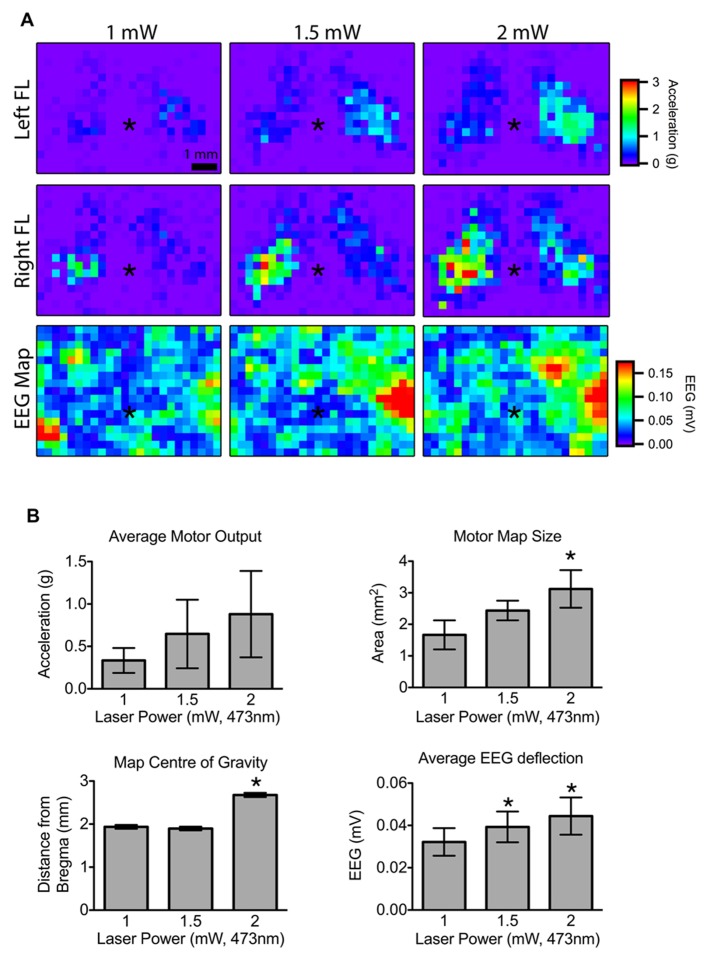
**Effect of laser power on motor output and cortical depolarization.(A)** Motor maps were generated from randomly interleaved trials of 1, 1.5, and 2 mW laser powers and the evoked movements from the left and right forelimbs (FL) were recorded. The corresponding EEG maps are shown below (asterisk “*” indicates bregma; each pixel = 300 μm). **(B)** Motor map size was significantly higher for 2 mW stimulation compared to 1 mW. The center of the 2 mW map was also significantly further from bregma compared to 1 or 1.5 mW maps. The magnitude of the average EEG deflection was significantly greater in the 1.5 and 2 mW maps relative to 1 mW (asterisk “*” indicates *p* <0.05 vs. 1 mW).

We next set out to determine the minimum laser power needed to reliably evoke contralateral forelimb movements during motor mapping. Given that motor output can vary significantly depending on the level of anesthesia, we interleaved light pulses of various powers within a single mapping session. A 2-mW laser pulse (5 ms duration) was sufficient to produce motor maps with well-defined borders (**Figure 3A**) and evoke cortical depolarization at the majority of the stimulated sites. Although the average motor output from each stimulation site was not significantly altered by laser power we found a significant increase in motor map size and a shift in map position with 2 mW light pulses (relative to 1 mW; *p* <0.0031; **Figure 3B**), indicating the recruitment of sites with higher photocurrent thresholds. The average magnitude of the EEG deflection also increased significantly with higher laser power (*p* = 0.0017). Laser pulses higher than 2.5 mW produced more bilateral movements (data not shown), which are also observed with suprathreshold ICMS stimulation (Brus-Ramer et al., 2009).

**FIGURE 4:**
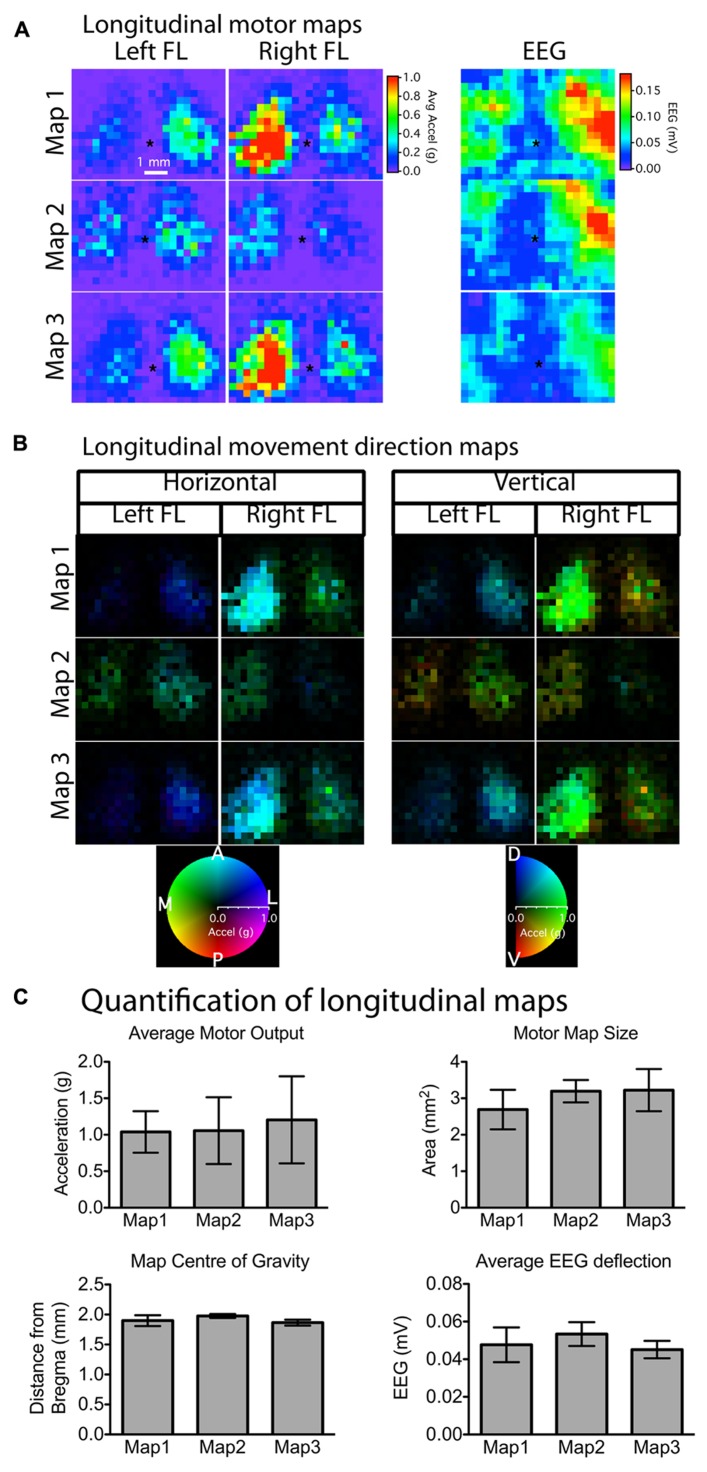
**Stability of motor and EEG maps across mapping sessions weeks apart. (A)** Motor and EEG maps generated weeks apart showed stability and reproducibility in terms of relative location (asterisk “*” indicates bregma) and size (number of responsive motor sites). Although there was noticeable variability in the absolute magnitude of movements between mapping sessions for individual animals **(A)**, average motor output, motor map size, as well as map location were not significantly different among any of the three time-points **(C)**. There was also no change in the magnitude of the average EEG deflection, suggesting that the preparation remained stable in terms of optical clarity and cortical excitability for the duration of the experiment. The majority of the evoked movements were in the anterior direction in the horizontal plane with a slight dorsal elevation in the vertical plane **(B)**. The brightness of each pixel in the movement direction maps represents the magnitude of the evoked movement, whereas the direction in the horizontal and vertical planes is indicated by the color of each pixel (each pixel = 300 μm).

**FIGURE 5:**
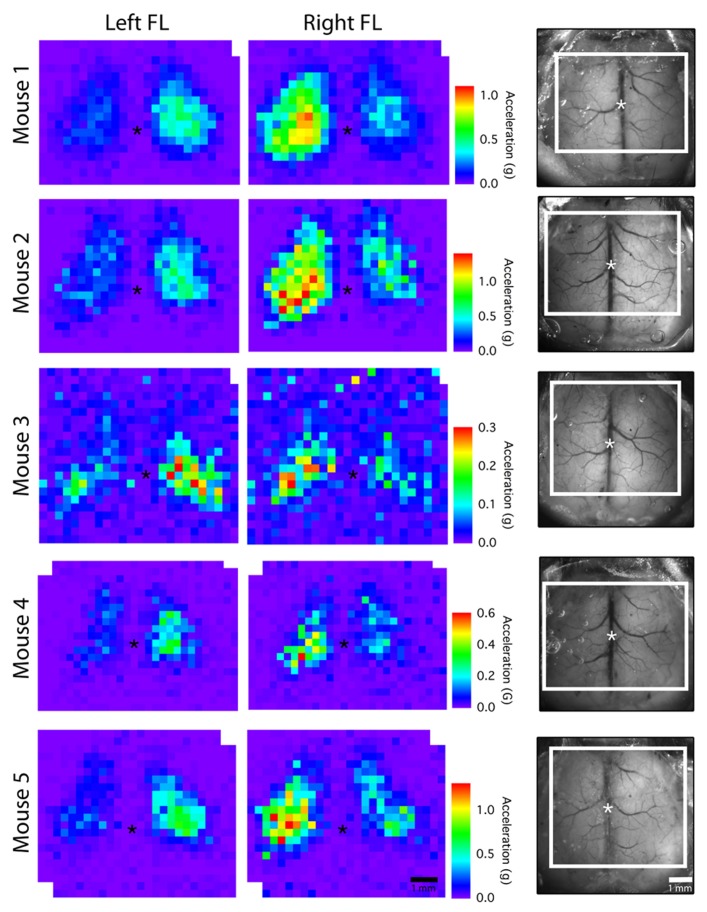
**Average motor maps for individual mice.** To assess inter-subject variability, motor output from the three mapping sessions was averaged to create an average motor map for each mouse. The dorsal view of the cranial window is shown on the right with the mapped region outlined in white (asterisk “*” indicates bregma; each pixel = 300 μm).

In order to assess the stability of our chronic window preparation over time we quantified the stability of motor and EEG maps over repeated mapping sessions spaced at least 1 week apart (**Figure 4A**). When we averaged all active pixels in the motor maps, there were no significant changes in average motor output, motor map size, or relative map location (center of gravity) over time (*p* > 0.2966, *n* = 5 mice; **Figure 4C**). Importantly, there was also no significant change in the magnitude of the EEG depolarization suggesting that light penetration and cortical excitability were equivalent during the three mapping sessions (*p* = 0.903). The direction of the evoked movements also showed clear consistency across time, as most movements were in the anterior direction in the horizontal plane, with a slight elevation in the vertical plane (**Figure 4B**). To show the qualitative features of motor map topography in all animals, we averaged the motor maps from three mapping sessions for each animal (**Figure 5**; *n* = 5 mice). By summing all active pixels (accelerations greater than five times the SD of baseline data) from each forelimb map we found the average center of gravity was located 1.65 mm lateral and 0.735 mm anterior from bregma, and the average area of the forelimb motor map was 3.02 mm^2^ (*n* = 5 mice).

### EEG RECORDINGS DURING MOTOR MAPPING

Implanting an EEG electrode next to the cranial window allowed us to record cortical depolarizations induced by light stimulation. Although our preparation included only one electrode on the lateral aspect of the right hemisphere, this was sufficient to record light-evoked depolarizations from both hemispheres (**Figure 2B**). The light-evoked depolarizations from the hemisphere opposite to the electrode were generally smaller; likely due to the limited receptive field of the recording electrode. We did not observe a photoelectric artifact (Cardin et al., 2010) as the tip of the electrode was just lateral to the area stimulated during mapping (**Figures 2C,D**). The magnitude of depolarization at each stimulation site was calculated by integrating the first 70 ms of the EEG trace after the stimulus and dividing by the integration period. Plotting the amplitude of this value as a heat-map over the cortex produced EEG maps, which could then be superimposed onto motor maps derived from the same stimulation trials (**Figure 2B**). By quantifying both cortical depolarization (EEG) and motor output we could confirm that non-motor sites forming the borders of a motor map are indeed activated by light stimulation but do not contribute to motor output. Chronic EEG recordings are typically performed in rats by threading stainless steel screws into the skull to serve as recording electrodes (Paz et al., 2012). This approach was not feasible in mice because the skull is much thinner compared to rats, and inserting the smallest available screws (#000) frequently caused cortical damage in our pilot experiments. In contrast, we found no evidence of tissue damage at the site of the EEG electrode in our mice with chronic windows (data not shown).

## DISCUSSION

LBMM was developed as a complementary technique to ICMS, with the advantage of being less invasive and thus repeatable in the same animal (Ayling et al., 2009; Hira et al., 2009). Here we describe the detailed methods for longitudinal motor mapping in mice implanted with bilateral chronic cranial windows and an EEG electrode. We demonstrate that evoked movements can be quantified in an automated fashion with lightweight accelerometers attached to the forelimbs and we monitor the stability of motor maps over several weeks.

The cranial window implantation described in the current study is adapted from a previous method where a smaller region of the skull (3 mm × 3 mm) was thinned, polished, and sealed with a layer of cyanoacrylide glue and a cover glass (Drew et al., 2010). In contrast to full craniectomies, where a piece of the skull is removed, the thinned-skull preparations are less invasive, producing less inflammation and gliosis (Yang et al., 2009; Drew et al., 2010; Parthasarathy et al., 2010). We found that thinned-skull preparations can be expanded to include the majority of the dorsal cortical surface of both hemispheres in mice (~20 mm^2^). In the context of motor mapping, this expanded area for stimulation provides several advantages. First, motor map size is heavily influenced by the level of anesthesia as well as laser power during stimulation (Harrison et al., 2012), and in previous studies under some circumstances motor maps extended to the borders of standard sized cranial windows (3.5 mm × 3.5 mm). Motor maps can also be significantly modulated in size and location by experimental manipulations such as CNS injury or application of pharmacological agents (Gharbawie et al., 2005; Young et al., 2011). The current preparation provides sufficient space to ensure that experimentally induced shifts in motor maps can be monitored accurately through time, without being impinged at the borders of the cranial window.

An additional challenge common to both electrode and LBMM is the difficulty in definitively and correctly identifying non-responsive cortical sites. The optimal dose of anesthesia varies significantly among animals, and physiological parameters such as heart rate are ineffective for monitoring anesthetic depth (Tandon et al., 2007). Therefore, the absence of evoked movement at a particular point may be due to the lack of motor connections from that site, or due to too much anesthetic. Having criteria in place to correctly distinguish between these two scenarios becomes especially important after perturbations to motor maps (such as cortical injury) where an increase in the number of non-responsive sites is expected. By interleaving stimulation sites from the contralateral hemisphere during mapping, our bilateral preparation provides a significant advantage as one hemisphere may serve as an intact control for anesthetic levels or experimental perturbations in the other hemisphere. In addition, the simultaneous EEG recording during mapping may also help to identify regions inadvertently damaged during surgical preparation or lacking sufficient opsin expression to induce depolarization.

This novel preparation also provides a non-invasive method for quantifying the magnitude and direction of optogenetically evoked movements during mapping of motor cortex. In contrast to ICMS protocols, where the minimum current needed to evoke a movement is determined at each stimulation site, we chose to apply the same power of light stimulation at each site and quantify the magnitude of the evoked movements from that stimulus. Attaching accelerometers to the forelimbs provides a convenient, non-invasive method for quantifying forelimb movements in an automated fashion, however, digit and wrist movements are not differentiated and may remain undetected. Given that movements of proximal muscles have lower current thresholds (Tennant et al., 2011), the laser power used in the current study may not be high enough to reliably evoke distal movements. Instead, laser power was optimized for reliably generating well-defined forelimb movement maps. We have used relatively short 5 ms pulses of light stimulation to evaluate the output of motor cortex. In rodents, longer trains of either ICMS or light stimulation have produced regionally complex movements that were analyzed with video recordings (Harrison et al., 2012; Bonazzi et al., 2013). Our automated tracking of the magnitude and direction of limb acceleration in a mouse preparation provides a convenient method for evaluating stimulation parameters that mimic the timescale of ethologically relevant behaviors (Graziano et al., 2002). Future experiments may also examine the topographical representation of stimulation evoked arm postures when the starting position of the limb is varied in the workspace of the animal, similar to work done in primates (Graziano, 2005).

The wires needed to power the accelerometers and to collect the data are an obvious limitation in our technique. Although it is possible that the weight of the device and the wired connections impose a constraint on the evoked movements, we positioned the wires consistently for all mapping sessions in order to minimize this confound.

Lastly, we would be remiss to not comment on the potential of applying this preparation to other forms of imaging to study sensory-motor interactions. Thin-skull preparations provide sufficient optical access for two-photon imaging (Drew et al., 2010), and recent advances have made two-photon activation of channelrhodopsin possible (Prakash et al., 2012). Combining these two methods *in vivo* would provide a powerful preparation for fine scale activation of motor circuits.

## Conflict of Interest Statement

The authors declare that the research was conducted in the absence of any commercial or financial relationships that could be construed as a potential conflict of interest.
